# Supplementary material for: Bias in Zipf’s law estimators
Source: Sci Rep. 2021 Aug 27;11:17309. doi: 10.1038/s41598-021-96214-w (PMC8397718; doi:10.1038/s41598-021-96214-w)
Supplement: Supplementary file 1 — Supplementary Information. [file 41598_2021_96214_MOESM1_ESM.pdf]

Supplementary Information
Bias in Zipf's Law Estimators

Charlie Pilgrim[1,*] **and Thomas T Hills**[2,3]

[1]The University of Warwick, Mathematics for Real-World Systems Centre for Doctoral Training, Coventry, CV4 7AL, UK
[2]The University of Warwick, Department of Psychology, Coventry, CV4 7AL, UK
[3]The Alan Turing Institute, British Library, 96 Euston Road, London, NW1 2DB
[*]charlie.pilgrim@warwick.ac.uk

Computational Complexity

The general likelihood for inferring probability distributions from rank-frequency data is given in the main paper as

$$p(\boldsymbol{n}|\boldsymbol{\theta}) = \sum_{\boldsymbol{s} \in S(W)} \prod_{r_p}^{W} p(x_{r_p})^{\boldsymbol{n}(\boldsymbol{s}(r_p))} \tag{1}$$

The number of terms in the likelihood function scales as $O(W!)$, so that naive computation of the likelihood is impractical even at $W \approx 10$. When analysing Zipf's law for words in a book W represents the writer's vocabulary. Even considering a lower bound for W as the number of unique words in a book, $W > 1000$ so that the likelihood is extremely computationally expensive using a naive algorithm. Here we will explore how to make this computation more efficient.

The full likelihood function (equation 1) is equivalent to the calculation of the permanent of a matrix with entries $a_{ij} = p(x_j)^{\boldsymbol{n}(i)}$.

$$A = \begin{bmatrix} p_1^{\boldsymbol{n}(1)} & p_2^{\boldsymbol{n}(1)} & \cdots & p_W^{\boldsymbol{n}(1)} \\ p_1^{\boldsymbol{n}(2)} & p_2^{\boldsymbol{n}(2)} & \cdots & p_W^{\boldsymbol{n}(2)} \\ \vdots & \vdots & \ddots & \vdots \\ p_1^{\boldsymbol{n}(W)} & p_2^{\boldsymbol{n}(W)} & \cdots & p_W^{\boldsymbol{n}(W)} \end{bmatrix} \tag{2}$$

$$\mathscr{L}(\boldsymbol{\theta}|\boldsymbol{n}, M)) = per(A) \tag{3}$$

The permanent is similar to the determinant, with the difference that the negative signs in the Laplace expansion formula for the determinant are all positive[1]. A well known algorithm for exactly computing the permanent of a matrix is Ryser's algorithm[2,3] with complexity $O(W2^W)$. The exact computation of the permanent is thought to be $\#P$-hard[4,5], so that no polynomial algorithm exists if $P \neq NP$. A polynomial time approximation algorithm for the permanent of a non-negative matrix (as our matrix is), was discovered by Jerrum et al[6], with complexity $O(W^{10})$. These algorithms are improvements on the naive case but are still prohibitively computationally expensive for the use case of a text corpora with a vocabulary of $W > 1000$.

We investigated a method of reducing the computational complexity of Ryser's algorithm (in our case) by several orders of magnitude by considering tied empirical ranks, which are equivalent to repeated columns in the matrix A. This can be done but the computation time remains extremely prohibitive. A lower bound to an estimate of the computational complexity using this technique would be $O(F2^F)$, where F is the number of unique empirical counts, as the computation would be at least as complex as computing the permanent of a matrix of the unique columns. This would remain prohibitively computationally expensive for real world data sets. The slim hope that remains is to use the structure and symmetry of the matrix to find some shortcut or a reasonable approximation, we leave this as an open question.

Approximate Bayesian Computation Regression with Mean Log

Approximate Bayesian computation is a technique for approximating posterior distributions without having to calculate a likelihood function[7–9]. Instead, we simulate data, $\boldsymbol{n}_i$, from possible parameters, λ_i, and observe how close that simulated data is to the empirical data (using a distance measure $\rho(\boldsymbol{n}_i, \boldsymbol{n}_{obs})$). By looking at the behaviour of simulated data with close distances, we can approximate the posterior distribution, $p(\lambda|\boldsymbol{n}_{obs})$.

In order to use ABC to we need a way to measure the "distance" between two data sets. A common technique is to summarise the data sets with a summary statistic, $S(\boldsymbol{n})$, and define the distance as the difference between those, $\rho(\boldsymbol{n}_i, \boldsymbol{n}_{obs}) = S(\boldsymbol{n}_i) - S(\boldsymbol{n}_{obs})$[7,10]. A good summary statistic will capture a lot of information relevant to the likelihood function so that $p(\lambda|\boldsymbol{n}) \sim p(\lambda|S(\boldsymbol{n}))$. With rank-frequency distributions, the mean of the logs of the observations is of a similar form to the likelihood function derived in the main paper. Through experiment this statistic was found to be a good candidate summary statistic.

$$S_i = \sum_{r_e=1}^{W} \boldsymbol{n}_i(r_e) log(r_e) \tag{4}$$

There are several flavours of ABC[9,10]. Here we use the regression method[8,9,11]. We only consider distances within some tolerance, ε, of the observed data, i.e. $|S(\boldsymbol{n}_i) - S(\boldsymbol{n}_{obs})| < \varepsilon$. The regression method has advantages over the rejection method that it is computationally more efficient and does not require careful tuning of the tolerance[8]. The key assumption is a linear approximation within the tolerance region:

$$\lambda_i = \beta S(\boldsymbol{n}_i) + \alpha + \phi_i \tag{5}$$

Assuming that ϕ has an invariant distribution within this tolerance region, we can find estimates $\hat{\beta}$ and $\hat{\alpha}$ using ordinary least squares regression. To estimate the posterior we are interested in $p(\lambda|S(\boldsymbol{n}_{obs}))$, which can be estimated by translating the data points along the regression line.

$$\lambda_i^* = \lambda_i - \hat{\beta}(S(\boldsymbol{n}_i) - S(\boldsymbol{n}_{obs})) \tag{6}$$

The frequency histogram of these translated points will be approximately proportional to the likelihood function. The histogram can be smoothed using a kernel density estimate and the mode taken to find the maximum likelihood estimator. The process is summarised in Figure 1.

ABC Regression Results

Rank-frequency data was generated ($N = 10000$) from an unbounded power law with exponents ranging from 1 to 2. For each generated data set, the exponent was estimated using a) Clauset et al's estimator and b) ABC. This was repeated 100 times to find the mean bias and variance. The ABC method has much lower bias and similar variance to Clauset et al's method, (Figure 2).

We also looked at changing sample size. Rank-frequency data was generated with $\lambda = 1.1$ and varying sample size up to $N = 1000000$. Clauset et al's estimator shows positive bias at all values of N, although it decreases with large N. ABC regression shows much less bias at all tested values of N. The variance of ABC regression is higher for $N \lessapprox 1000$. Overall the variance is still very low, and is insignificant compared to the positive bias showed by Clauset et al's estimator (Figure 3).

Overall ABC regression with the mean log as a summary statistic shows much less bias and similar variance to Clauset et al's estimator, when applied to data generated from a Zipfian probability distribution.

References

1. Agrawal, M. Determinant versus permanent (European Mathematical Society, 2006).
2. Ryser, H. J. *Combinatorial mathematics*, vol. 14 (American Mathematical Soc., 1963).
3. Glynn, D. G. The permanent of a square matrix. *Eur. J. Comb.* **31**, 1887–1891, DOI: 10.1016/j.ejc.2010.01.010 (2010).
4. Valiant, L. G. The complexity of computing the permanent. *Theor. computer science* **8**, 189–201 (1979).
5. Scott, A. A linear-optical proof that the permanent isP-hard. *Proc. R. Soc. A.* **467**, 3393–3405, DOI: 10.1098/rspa.2011.0232 (2011).

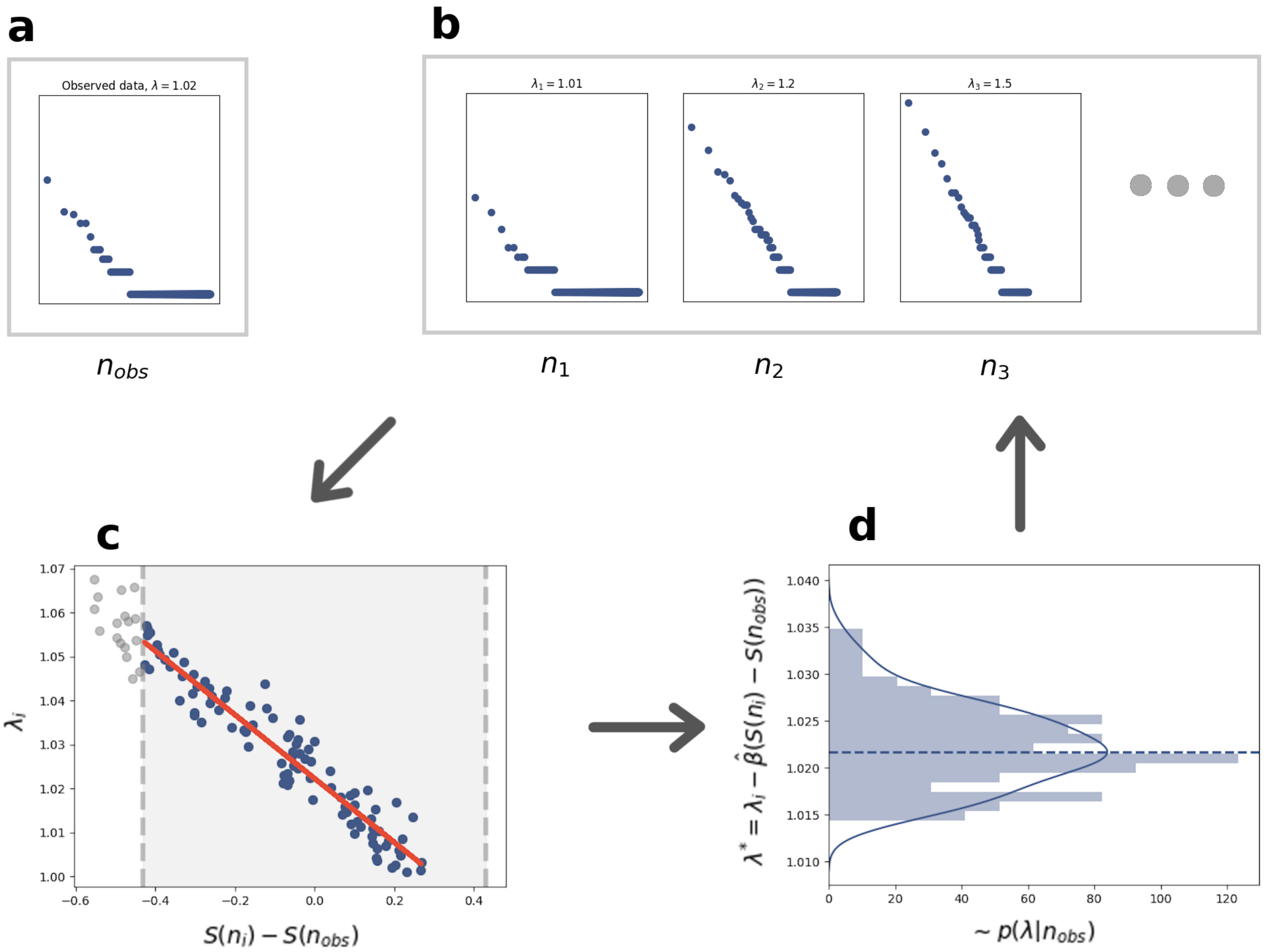

Figure 1. Approximate Bayesian computation regression with the mean log. ABC proceeds as shown. a) A summary statistic $S(\boldsymbol{n})$ is calculated from the observed data. b) Parameters are sampled from a uniform distribution. For each parameter, λ_i a set of data, $\boldsymbol{n}_i$, is generated, and a summary statistic, $S(\boldsymbol{n}_i)$, is calculated. c) A tolerance is chosen to accept a given proportion, P_ε, of the simulations with close summary statistics to the observed data, shown as the shaded region. A linear regression is fit to the accepted simulation results. d) The accepted parameters are adjusted along the regression line to $S(\boldsymbol{n}_i) = S(\boldsymbol{n}_{obs})$. The histogram of these corrected parameter values approximates the likelihood function. A kernel density estimate is used to smooth the likelihood and find the maximum likelihood estimate for λ. Here the initial data was generated with $\lambda = 1.02$ and the maximum likelihood estimator was $\hat{\lambda} = 1.023$, this is a typical result. Figure idea adapted from[7] and[9].

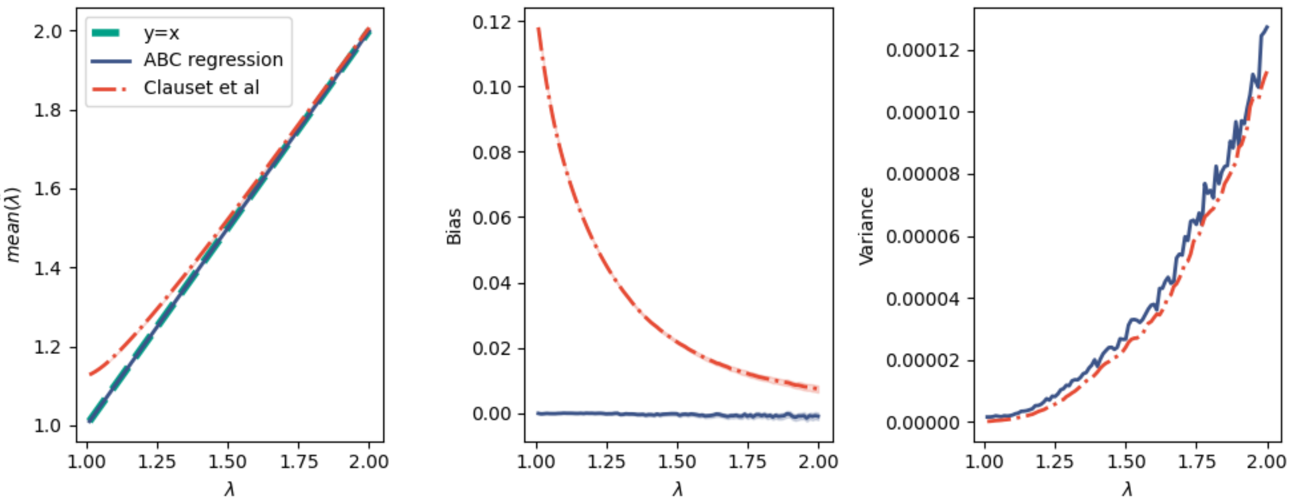

Figure 2. Bias in ABC regression (blue solid line) vs Clauset et al's estimator (red dashed line) for unbounded power laws. Rank-frequency data was generated with $N = 10,000$ for 100 values of λ between 1.01 and 2. This was run 100 times. The left figure shows the known λ against the mean estimated $\hat{\lambda}$ over 100 runs. The central figure shows the mean bias (the difference between the mean estimated $\hat{\lambda}$ and λ) with a shaded 68% confidence interval. The right figure shows the variance of the estimators. The ABC estimator has much less bias and similar variance to Clauset et al's estimator.

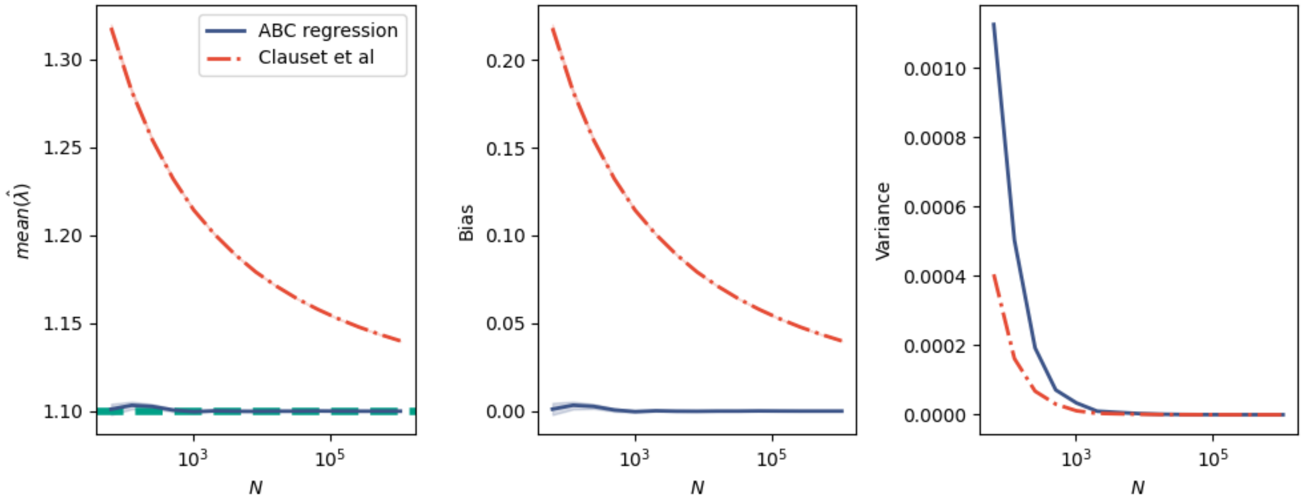

Figure 3. Bias in ABC regression (blue solid line) vs Clauset et al's estimator (red dashed line) for unbounded power laws. Rank-frequency data was generated for $\lambda = 1.1$ with varying sizes, N. This was run 100 times. The left figure shows the known λ against the mean estimated $\hat{\lambda}$. The centre figure shows the mean bias, with a 68% confidence interval shaded. The right figure shows the variance of the estimators. The ABC estimator has much smaller bias and similar variance to Clauset et al's estimator.

6. Jerrum, M., Sinclair, A. & Vigoda, E. A polynomial-time approximation algorithm for the permanent of a matrix with nonnegative entries. *J. ACM (JACM)* **51**, 671–697 (2004).

7. Sunnåker, M. *et al.* Approximate Bayesian Computation. *PLoS Comput. Biol.* **9**, e1002803, DOI: 10.1371/journal.pcbi.1002803 (2013).

8. Beaumont, M. A., Zhang, W. & Balding, D. J. Approximate Bayesian Computation in Population Genetics. *Genetics* **162**, 2025–2035 (2002).

9. Csilléry, K., Blum, M. G. B., Gaggiotti, O. E. & François, O. Approximate Bayesian Computation (ABC) in Practice. *Trends Ecol. Evol.* **25**, 410–418, DOI: 10.1016/j.tree.2010.04.001 (2010).

10. Beaumont, M. A. Approximate Bayesian Computation in Evolution and Ecology. *Annu. Rev. Ecol. Evol. Syst.* **41**, 379–406, DOI: 10.1146/annurev-ecolsys-102209-144621 (2010).

11. Leuenberger, C. & Wegmann, D. Bayesian Computation and Model Selection Without Likelihoods. *Genetics* **184**, 243–252, DOI: 10.1534/genetics.109.109058 (2010).
